# The Multiple Roles of B Cells in the Pathogenesis of Sjögren’s Syndrome

**DOI:** 10.3389/fimmu.2021.684999

**Published:** 2021-06-08

**Authors:** Wenhan Du, Man Han, Xiaoxia Zhu, Fan Xiao, Enyu Huang, Nan Che, Xiaopo Tang, Hejian Zou, Quan Jiang, Liwei Lu

**Affiliations:** ^1^ Department of Pathology and Shenzhen Institute of Research and Innovation, The University of Hong Kong, Hong Kong, China; ^2^ Division of Rheumatology, Guang’anmen Hospital, China Academy of Chinese Medical Sciences, Beijing, China; ^3^ Department of Rheumatology, Huashan Hospital and Fudan University, Shanghai, China; ^4^ Chongqing International Institute for Immunology, Chongqing, China; ^5^ Department of Rheumatology, The First Affiliated Hospital of Nanjing Medical University, Jiangsu, China

**Keywords:** primary Sjögren’s syndrome, B cells, pathogenesis, treatment, regulatory functions

## Abstract

Primary Sjögren’s syndrome (pSS) is a chronic autoimmune disease characterized by lymphocytic infiltration and tissue destruction of exocrine glands such as salivary glands. Although the formation of ectopic lymphoid tissue in exocrine glands and overproduction of autoantibodies by autoreactive B cells highlight the critical involvement of B cells in disease development, the precise roles of various B cell subsets in pSS pathogenesis remain partially understood. Current studies have identified several novel B cell subsets with multiple functions in pSS, among which autoreactive age-associated B cells, and plasma cells with augmented autoantibody production contribute to the disease progression. In addition, tissue-resident Fc Receptor-Like 4 (FcRL4)^+^ B cell subset with enhanced pro-inflammatory cytokine production serves as a key driver in pSS patients with mucosa-associated lymphoid tissue (MALT)-lymphomas. Recently, regulatory B (Breg) cells with impaired immunosuppressive functions are found negatively correlated with T follicular helper (Tfh) cells in pSS patients. Further studies have revealed a pivotal role of Breg cells in constraining Tfh response in autoimmune pathogenesis. This review provides an overview of recent advances in the identification of pathogenic B cell subsets and Breg cells, as well as new development of B-cell targeted therapies in pSS patients.

## Introduction

Primary Sjögren’s syndrome (pSS) is a common systemic autoimmune disease, which mainly affects the lacrimal and salivary glands, resulting in dry eyes and dry mouth. However, the extra-glandular manifestations occur in 30 to 40% of pSS patients, involving lung, heart, kidney, nervous system and lymphoproliferative disorders (1, 2). Although the etiology of pSS remains unclear, numerous studies have demonstrated that both T and B cells are the major populations for pro-inflammatory cytokine production and autoantibody secretion with critical involvement in pSS pathology (3). A recent multiple-centre study reported that pSS patients in China had higher positive rates of anti-nuclear antibody (ANA) and anti-Sjögren’s syndrome-related antigen A (anti-SSA) antibodies than those of patients in Europe and America, indicating that disease heterogeneity among pSS patients in different regions (4). Growing evidence indicates that B cells play predominant roles in the pathogenesis of pSS patients with ectopic germinal center-like structures in the exocrine glands and systemic extra-glandular manifestations (5, 6). It has been well recognized that autoreactive B cells and plasma cells contribute to the development of pSS by producing various autoantibodies, including ANA, anti-SSA and anti-Sjögren’s syndrome type B (anti-SSB) antibody (7, 8). Recent studies have revealed other functions of B cells such as cytokine production (9, 10) and antigen presentation (11) in the pathogenesis of pSS. Increasing evidence indicates the functional diversities of B cell subsets in both immunity and autoimmune pathogenesis (12). In particular, Breg cells with different phenotypes have been reported to be involved in the development of pSS (13–16). Breg cells exerted their regulatory functions by producing diverse regulatory cytokines and effector molecules, such as IL-10, IL-35 and Granzyme B (GrB). Although B cell targeted therapy is among the most promising therapeutic approaches to various autoimmune diseases, its efficacy in treating pSS patients remains to be further validated. In this review, we discuss the multiple functions of B cell subsets in pSS development and emerging B cell-targeted therapies.

## Pathogenic B Cell Subsets

### Fc Receptor-Like 4 (FcRL4)^+^ B Cells

FcRL4^+^ B cells are tissue-resident memory B cells that express Fc Receptor-Like 4 (FcRL4). As a member of Fc Receptor-Like proteins family, FcRL4 is mainly expressed by the B-cell lineage (17). FcRL4 is found to dampen B cell receptor-mediated signaling and proliferation, which plays an essential role in regulating B cell activation and differentiation. In 2005, Max Cooper and colleagues first described these B cells in human tonsils (18). Subsequent studies confirmed that FcRL4^+^ B cells were mainly localized in the sub-epithelial region of lymphoid tissues but rarely in spleen and lymph nodes (19, 20). FcRL4^+^ B cells are expanded in the inflamed tissues of patients with pSS (21, 22). Moreover, enriched FcRL4^+^ B cells are detected in salivary glands of pSS patients (21). Further studies on the comparison of FcRL4^+^ B cells with chemokine receptor CCR5 expression profiles from the parotid glands of pSS patients provide a perspective for understanding their migratory ability to the corresponding chemokines CCL3 and CCL5 produced by ductal epithelial cells and their infiltration in the inflamed glands (22, 23). In addition, studies with gene transcription analysis suggest that parotid FcRL4^+^ B cells with upregulated *CXCR3* expression may enhance their migration (22). Notably, compared with patients without lymphoma, the percentages of FcRL4^+^ B cells are significantly increased in pSS patients with MALT-lymphomas, indicating that these B cells may contribute to the development of B cell lymphoma in pSS patients (21).

**Figure 1:**
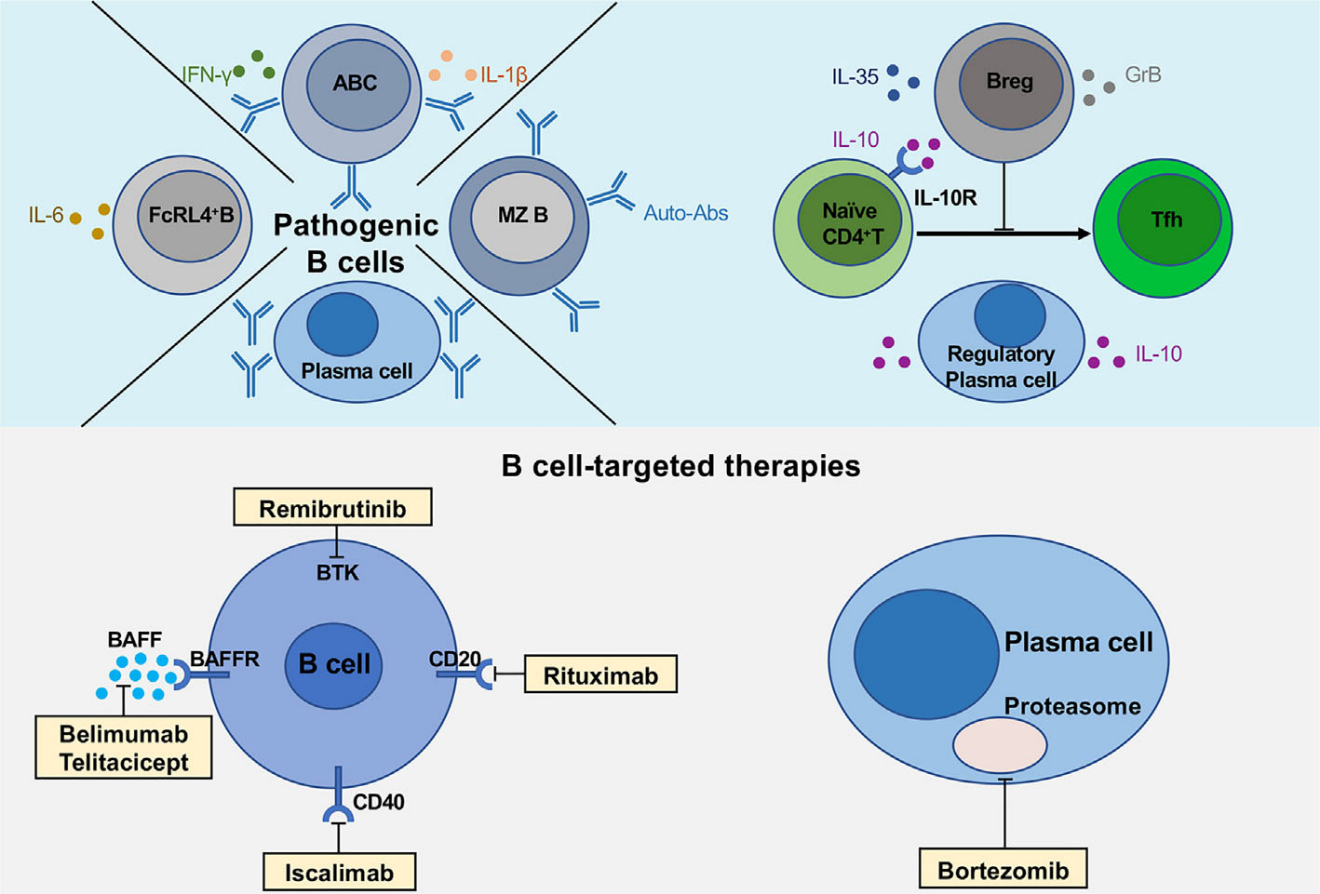
Multiple functions of B cells and novel B cell-targeted therapies in pSS. The upper part of the figure shows the pathogenic B cell subsets (FcRL4^+^ B cells, age-associated B cells and plasma cells), regulatory B cells and plasma cells secreting various cytokines, IL-10, IL-35 and GrB. The lower part describes the multiple B cell-targeted therapies, consisting of direct and indirect B cell depletion, plasma cell depletion.

Lines of evidences suggest that FcRL4^+^ B cells play a pathogenic role in the development of pSS (21, 22) (**Table 1**). The percentages of parotid FcRL4^+^ B cells are positively correlated with the numbers of lymphoepithelial lesions in pSS patients (21). Additionally, FcRL4^+^ B cells produce multiple pro-inflammatory cytokines. *IL-6* gene is significantly upregulated in FcRL4^+^ B cells from pSS patients, suggesting that FcRL4^+^ B cell is a pro-inflammatory B cell subset in autoimmune diseases (22) (**Figure 1**). Interestingly, a recent study with transcription analysis of gene expression has revealed that FcRL4^+^ B cells highly express *ITGAX* (CD11c) and *TBX21* (T-bet) genes (22), similar to the gene expression pattern detected in CD11c^+^T-bet^+^ B cells (24, 25). Although both B cell subsets displayed similar patterns of downregulated BCR signaling and enhanced TLR signaling, they may possess different capacities in differentiating into antibody-secreting cells (ASCs) since the lack of transcription factors Blimp1 and IRF-4 in FcRL4^+^ B cells may reduce their ability to differentiate into ASCs (21). It has been reported that the treatment with Rituximab reduces the number of parotid gland FcRL4^+^ B cells and restores the glandular epithelium in pSS patients (21). As the first and most widely studied B cell-targeted therapeutic agent, Rituximab depletes mature B cells effectively, lasting four to twelve months (47). In addition to FcRL4^+^ B cells, circulating Tfh cells and Th17 cells are also reduced by Rituximab accompanied by decreased serum IL-21 and IL-17 levels (48). Thus, further elucidation of the pathogenic mechanisms of FcRL4^+^ B cells may facilitate the development of novel therapeutic strategies for targeting this B cell subset in pSS.

**Table 1:** Pathogenic and regulatory B cell subsets in pSS.

B cell subset	Markers	Functions	Potential therapy
**FcRL4^+^ B cell**	FcRL4, CCR5, *CXCR3, ITGAX*, *TBX21* (22, 23)	Pathogenic	Rituximab (21)
**Age-Associated B cell**	CD11c, T-bet, CXCR5, CD21, CD23 (24–26)	Pathogenic	Belimumab (27) Telitacicept (28) Remibrutinib (29, 30) Iscalimab (31), Abatacept (32)
**Transitional B cell**	CD21, IgD, IgM (33)	Pathogenic	
**Marginal Zone B cell**	CD21, CD23, IgD (34)	Pathogenic	Rituximab
**Memory B cell**	CD27, CXCR4, CXCR5 (35)	Pathogenic	
**Plasma cell**	CD138, CD27, CD38, Bcl-2 (36–39)	Pathogenic	Bortezomib (40)
**IL-10^+^ Breg**	CD24, CD38, CD1d, CD5, IL-10 (41, 42)	Protective	
**GrB^+^ Breg**	CD5, GrB (43)	Protective	
**IL-35^+^ Breg**	CD138, TACI, CXCR4, IL-35 (44, 45)	Protective	
**Regulatory Plasma Cell**	LAG-3, CD138 (46)	Protective	

### CD11c^+^Age-Associated B Cells

CD11c^+^Age-associated B cells (ABCs) were firstly reported by two independent research groups in 2011 (24, 25). CD11c^+^ABCs are characterized by phenotypic markers including CD11c^+^, CD11b^+^, CD21^-/low^, CD23^low^ (24, 25), which are notably expanded in the peripheral blood of patients with several autoimmune diseases, including systemic lupus erythematosus (SLE) (49, 50), RA (25) and multiple sclerosis (MS) (51). In pSS patients, CD11c^+^ABCs infiltrated in parotid glands have been identified with double staining for CD11c and Pax5 (22). CD11c^+^ABCs express high levels of the transcription factor T-bet, exhibiting a distinct phenotype from other B cell subsets (24–26). T-bet is a transcription factor that has been considered as a hallmark of Th1 cells. However, recent studies have revealed that T-bet is also expressed in certain B cells with co-expression of CD80 and CD86, suggesting that these B cells are potent antigen-presenting cells (52) (**Table 1**). CD11c^+^ABCs appear to be recruited into inflamed tissues *via* specific chemokine-chemokine receptor axis. CD11c^+^ABCs with low levels of CXCR5 and CCR7 expression are localized outside the B cell follicles (53). Experiments with adoptive transfer of CD11c^+^ABCs have confirmed their location at the boundary region between T cell and B cell zones, which is very similar to the location of interfollicular large B cells that are found in the T cell-rich area of secondary lymphoid organs and ectopic lymphoid structures in pSS patients (54, 55). The interfollicular large B cells express high levels of Activation-induced cytidine deaminase and are associated with B cell lymphoma in patients with pSS (56). CD11c^+^ABCs can secrete various cytokines, including IL-1β, IL-6, IFN-γ and IL-10 (**Figure 1**).

Available data suggest that CD11c^+^ABCs may be derived from different cell compartments in healthy people and SLE patients. It has been reported that CD11c^+^ABCs are mainly CD27^+^IgD^-^ switched memory B cells in healthy people (57). However, CD11c^+^ ABCs in SLE patients are enriched in CD27^-^IgD^-^double negative (DN) B cell population that can be further divided into two subsets, DN1 and DN2, according to the chemokine receptor CXCR5 expression (49). IFN-γ can trigger naïve B cell precursors to become activated naïve or DN1 B cells with high levels of T-bet expression (58). CD27^-^IgD^-^CXCR5^-^ (DN2) B cells are capable of differentiating into plasma cells induced by IL-21 in lupus patients. Frequencies of DN2 B cell are associated with lupus-related autoantibodies, including antibodies to dsDNA, nucleosome, histones and chromatin. Thus, autoreactive CD11c^+^ABCs may participate in the pathogenesis of lupus by secreting high titers of autoantibodies derived from an extrafollicular response. Moreover, type I interferon produced by plasmacytoid dendritic cells can promote plasmablasts to secrete anti-dsDNA antibodies from extrafollicular responses (59). It has been shown that depletion of CD11c^+^ABCs greatly reduces autoantibody levels and disease manifestations in lupus mice, which further confirms that CD11c^+^ABCs play a pathogenic role in the development of lupus (25). In pSS patients, the functional features of CD11c^+^ABCs remain largely unclear. A recent study has demonstrated that upregulated IL-21 signaling pathway in salivary glands of pSS patients is associated with enriched B cells and increased disease activity (60). Moreover, expanded IL-21^+^ Tfh cells in SS patients are associated with ectopic lymphoid structures and MALT-lymphomas (61). Given the accumulating evidence on a vital role of IL-21 in regulating CD11c^+^ABCs in lupus patients, it is likely that enhanced IL-21 signaling in salivary gland may promote CD11c^+^ABCs functions in the pathogenesis of pSS.

Although it is currently unclear whether CD11c^+^ABCs can be depleted by anti-CD20 or anti-CD19 therapies, targeting CD11c^+^ABCs is an appealing approach for the treatment of patients with autoimmune diseases. Previous studies reported elevated levels of both B-cell activating factor (BAFF) and A proliferation-inducing ligand (APRIL) in serum and saliva of pSS patients compared with healthy control (62, 63). Further immunohistochemical staining and transcript analysis reveal that the primary sources of BAFF are infiltrated T and B cells as well as ductal epithelial cells (64). Although the survival factors for CD11c^+^ABCs remain to be determined, CD11c^+^ABCs express high levels of BAFF receptor (BAFFR), intermediate densities of Transmembrane activator and CAML interactor (TACI) and minimal B-cell maturation antigen (BCMA) in lupus patients (50). A recent study has shown that anti-BAFF treatment with Belimumab reduces circulating CD11c^+^ABCs in SLE patients (27). Hence, these findings suggest that BAFF may regulate the survival of CD11c^+^ABCs in the local tissues of pSS patients. Recently, Belimumab has been evaluated in an open-label trial of 30 patients with pSS (65). The results showed that sixty percent of pSS patients achieved the primary endpoint whereas the disease activity index was significantly decreased at week 28. Considering that CD11c^+^ABCs may participate in the pathogenesis of pSS, it is possible that Belimumab can ameliorate the disease progression by depleting ABCs in pSS. Another promising therapy for targeting CD11c^+^ABCs is Telitacicept, which is a novel TACI-Fc fusion protein that binds to BAFF and APRIL (28). A phase 2b clinical trial of Telitacicept has reported a statistically significant difference in the clinical response rate between Telitacicept group (79.2%) and the placebo group (32%) (66). Currently, a phase 2 clinical study to evaluate Telitacicept effects on pSS patients is underway (Clinical Trials: NCT04078386). Furthermore, the blockade of cytokines such as IFN-γ, type I interferon or IL-21 may be promising strategies to target CD11c^+^ABCs since these cytokines are critically involved in triggering B cell activation and differentiation (50, 58). Emerging evidence reveals that enhanced activity of Bruton’s tyrosine kinase (Btk) in peripheral blood B cells promotes IL-21-mediated signaling pathway by inducing nuclear phosphorylated STAT1 levels in patients with autoimmune disease (29, 30). Hence, blockade of Btk (Remibrutinib) may possibly inhibit the pathogenic function of CD11c^+^ABCs. It has been recently reported that both frequencies and numbers of ABC with high levels of CD11c and T-bet expression are significantly reduced in aged CD154 (CD40L)-deficient mice compared to controls, indicating that CD40-CD40L interaction is essential for ABC generation (31). Thus, the blockade of CD40-CD40L interaction with Iscalimab may also represent a potential therapeutic strategy for targeting CD11c^+^ABCs in the treatment of pSS. CD21^-/low^ B cells, a subset of CD11c^+^ABCs, are found to be associated with lymphoproliferation in pSS patients (67), in which both percentages and absolute numbers in pSS patients are significantly increased when compared with healthy controls. Moreover, CD21^-/low^ B cells are also expanded in other autoimmune diseases, such as SLE (68) and RA (69). Similar to FcRL4^+^ B cells, CD21^-/low^ B cells highly express CD11c and FcRLs, including FcRL2 and FcRL3. These B cells exhibit profound activation defects when they are triggered by BCR and CD40 but could be activated by the stimulation of TLRs (TLR3, TLR7 and TLR9) (67). Furthermore, CD21^-/low^ B cells express high levels of co-stimulatory molecules CD80 and CD86, which enable them to act as antigen-presenting cells for T cell cognate interaction (70). CD21^-/low^ B cells promote the progression of pSS by secreting high-affinity autoantibodies, including anti-cytoplasmic and anti-nuclear autoantibodies (67). As a selective co-stimulation modulator, Abatacept directly binds to CD80 and CD86, which may reduce the antigen-presenting function of CD21^-/low^ B cells (32).

### Transitional B Cells

Transitional B cells are the B cell subset newly emigrated from the bone marrow to the secondary lymphoid organs. Upon activation by cognate antigens, CD21^+/-^IgD^+/-^IgM^+/-^ transitional type-1 B cells can differentiate into CD21^+^IgD^+^IgM^+^ type-2 B cells. It has been recently reported that new transitional CD21^low^CD10^+^IgM^hi^CD27^-^ B cells expressing polyreactive antibodies are increased in the peripheral blood of pSS patients (33) (**Table 1**). This observed increase in transitional B cells may reflect the defective central B cell tolerance in pSS patients. Moreover, transitional type-2 B cells are expanded in the ectopic germinal center-like structures of salivary glands in pSS patients (71). The available findings suggest that transitional B cells may drive the local tissue functional impairment and inflammation by producing antibodies.

### Marginal Zone B Cells

Recent studies have reported increased marginal zone B cells (MZ B) in patients with pSS (71) and mice with SS-like symptoms (8, 72). Phenotypic analysis shows that MZ B cells express high levels of CD21, but low levels of CD23 and IgD (34) (**Table 1**). In pSS patients, MZ B cells are found to be accumulated in the salivary glands and contribute to the glandular destruction by producing autoantibodies (71). In BAFF transgenic mice, expansion of MZ B cells is observed in both spleen and salivary glands while depletion of MZ B cells significantly reduces the infiltrations in the salivary glands (72). In another SS mouse model, IL-14 alpha transgenic mice with specific elimination of MZ B cells exhibit normal saliva secretions and histology of salivary glands, suggesting that MZ B cells play an indispensable role in the pathogenesis of SS (8). Moreover, MZ B cells are closely involved in the development of non-Hodgkin’s B-cell lymphoma, one of the most severe complications, in pSS patients (73). In a clinical study, Rituximab has been used to treat pSS patients with marginal zone lymphomas, which represents a promising therapeutic option (74).

### Memory B Cells

Recent studies have suggested that memory B cells are also involved in the pathogenesis of pSS. Although CD27^+^ memory B cells are reduced in the peripheral blood, memory B cells are accumulated in the salivary glands of pSS patients (75) (**Table 1**). Notably, CD27^+^ memory B cells highly express chemokine receptors CXCR4 and CXCR5, which may facilitate the infiltration of memory B cells into the inflamed glands by the chemokines CXCL12 and CXCL13 derived from epithelial cells (35). Similar to transitional B cells, CD27^+^ memory B cells appear to promote the formation of ectopic germinal center-like structures in the exocrine glands of pSS patients (76).

### Plasma Cells

Compelling evidence indicates that plasma cells (PCs) contribute to the autoimmune pathogenesis by producing large amounts of autoantibodies (**Table 1**). It has been reported that the numbers of IgG but not IgA expressing CD138^+^ PCs in salivary glands are correlated with focus scores of lymphocytic infiltrations in pSS patients (36). A recent study has also reported that CD19^+^CD27^hi^ plasma cells in salivary glands are positively correlated with serum ANA titers in pSS patients (37). Consistently, Szyszko et al. have observed increased CD38^+^CD138^+^ plasma cells in pSS patients (38). Notably, certain infiltrated PCs show phenotypic characteristics of the long-lived plasma cells (LLPCs), which highly express Bcl-2 but not Ki67. In a spontaneous SS mouse model, PCs detected in the submandibular glands are mostly BrdU^-^ in 40-week-old mice, showing the key features of LLPCs (39). It has been suggested that salivary glands provide a unique microenvironment for the survival and maintenance of LLPCs. The salivary gland epithelial cells are found to produce IL-6, a pivotal cytokine that support the survival of LLPCs. Our recent studies have identified a novel function of IL-17 in maintaining the survival of LLPCs *via* p38-mediated Bcl-xL RNA stability in murine lupus (77). Interestingly, epithelial cells in the salivary gland also secrete BAFF and APRIL, which may promote the survival of LLPCs (78, 79). Thus, these key cytokines and other factors including CXCL12 and CD44 provide the survival niche for LLPCs and promote persistent antibody production in salivary gland during pSS development.

Bortezomib (BTZ), a proteasome inhibitor that induces plasma cell apoptosis, has been found to be effective in treating various autoantibody-mediated autoimmune diseases in mice (80–82). BTZ can eliminate both short- and long-lived plasma cells and ameliorate lupus nephritis in mice (83). Our recent studies have also shown that BTZ can suppress Th17 response and autoantibody production in mice with experimental Sjögren’s syndrome (ESS) (40) (**Figure 1**). Several clinical studies have reported that BTZ treatment significantly reduces the levels of autoantibodies and improves the symptoms in patients with autoimmune disease, including refractory pSS, refractory SLE and thrombotic thrombocytopenic purpura (84–91). Thus, further investigation on the functional characteristics of LLPCs in pSS will facilitate the identification of therapeutic candidates for targeting these B cells.

Recently, two randomized controlled trials using Rituximab for treating pSS patients have failed to achieve their primary endpoints (92, 93). Several possible reasons may explain the failure of these clinical trials. As an anti-CD20 monoclonal antibody, Rituximab may deplete B cells by antibody-dependent cell-mediated cytotoxicity, complement-dependent cytotoxicity and, to a lesser extent, direct signaling through CD20. However, the inhibitory FcγRIIb expression on target B cells may promote Rituximab internalization and contribute to drug resistance (94). Another potential mechanism for Rituximab resistance is CD46-mediated inhibition of complement activation as increased serum CD46 levels are detected in pSS patients (95, 96). Thirdly, long-lived plasma cells express no or very low levels of CD20 and produce large amounts of autoantibodies in pSS patients, for which Rituximab treatment fails to target. In pSS patients, infiltrated B cells and locally differentiated plasma cells reside in the salivary glands while salivary gland epithelial cells produce large amounts of cytokines or pro-inflammatory factors for B cell survival such as BAFF (78). A clinical case report has recently shown that anti-CD20 treatment followed by Belimumab exhibits a synergistic effect with dramatically reduced EULAR Sjögren’s syndrome disease activity index (ESSDAI) in pSS patients with refractory cryoglobulinemic vasculitis (97). Thus, further large clinical trials are needed to validate the efficacy of this sequential therapy in the treatment of pSS.

## Regulatory B Cell Subsets

Previous studies have demonstrated that Breg cells are a subset of B cells that can negatively regulate immune response and autoimmune inflammation (98–100). Accumulated data suggest that different Breg subsets may share overlapping surface markers. In 2010, an elegant study by Mauri and colleagues identified human Breg cells with a phenotype of CD19^+^CD24^hi^CD38^hi^ B cells (41). Other investigations (101) also show that IL-10-producing B cells in human blood are enriched within CD24^hi^CD27^+^ B cell population. Moreover, IL-10-producing B cells are identified among CD27^int^CD38^hi^ plasmablasts and exert their regulatory function during autoimmune pathogenesis in humans and mice (102). Up to date, many studies have demonstrated that Breg cells exert their inhibitory functions *via* various effector mechanisms in autoimmune diseases (103–105) (**Table 1**).

### IL-10-Producing Breg Cells

In the pathogenesis of pSS, both T and B cells are prominently involved in lymphocytic infiltration and tissue inflammation in salivary glands. In ESS mice induced by immunization with salivary gland protein, Th17 cells have been shown to play a key role in initiating autoimmune inflammation and disease progression (106). Moreover, studies by Fu et al. have revealed that the deficiency of Tfh cells attenuates autoantibody production and disease progression during ESS induction in *Bcl6* ^fl/fl^ *Cd4* ^Cre^ mice, highlighting a crucial role of Tfh cells in driving autoantibody responses and ESS progression (107). Our early studies have indicated that IL-10-producing Breg cells can potently suppress Th17 response and ameliorate collagen-induced arthritis (108). Recently, we have observed negative correlations between IL-10-producing Breg cells and Tfh cell response in both pSS patients and ESS mice (42). During pSS progression, gradually reduced Breg cell frequencies are accompanied with expanded Tfh cells along with increased disease activities. In culture, Breg cells suppressed human and murine Tfh cell differentiation by promoting STAT5 phosphorylation in IL-10-dependent manner (42). Together, these findings indicate that IL-10-producing Breg cells can restrain Tfh cell response during pSS development (**Figure 1**). Notably, adoptive transfer of Breg cells markedly suppressed Tfh cell response and alleviated disease progression in ESS mice, indicating the potential application of Breg cell transfer as cell therapy for pSS (42). Our previous studies have demonstrated that low levels of BAFF can induce the differentiation of IL-10-producing Breg cells (109). Further studies have revealed that BAFF can induce IL-10 production *via* activating TACI-mediated signal transduction in normal and chronic lymphocytic leukemia B cells (110). Thus, it remains to be investigated whether and how the blockade of BAFF with Belimumab treatment may affect the generation or maintenance of Breg cells in patients with pSS or other autoimmune diseases.

In pSS patients (16), multiple subtypes of IL-10-producing Breg cells with different markers including IgA, IgG and IgM are identified. Interestingly, percentages of IgA-expressing Breg cells are higher in pSS patients than that of healthy individuals. However, frequencies of IgG-expressing Breg cells are reduced in pSS patients while IgM-expressing Breg cells are similar between pSS patients and controls. It has been reported that APRIL can induce naïve human B cells to IgA^+^ IL-10-producing Breg cells (111). Hence, elevated APRIL levels in SS patients may promote the formation of IgA-expressing Breg cells. Further characterization of Ig-expressing Breg cells will provide new insight in understanding their functional implications in the pathogenesis of pSS.

### GrB-Producing Breg Cells

In addition to IL-10, other effector molecules or cytokines are also involved in the regulatory functions of B cells. GrB belongs to the serine protease family that triggers target cell apoptosis with perforin. An early study revealed that GrB inhibits CD4^+^ T cell proliferation *via* a perforin-independent manner (112). The frequencies of GrB-producing Breg cells may vary among different autoimmune diseases. In pSS patients, GrB-producing CD19^+^CD5^+^ B cells with higher IL-21 receptor (IL-21R) expression are increased in the peripheral blood, along with expanded IL-21-producing invariant NKT cells (43). However, reduced GrB-producing Breg cells with lower IL-21R are observed in RA patients and negatively correlated with disease activity (113). Similarly, the frequencies of GrB-secreting Breg cells are decreased in SLE patients, especially in patients with lupus nephritis (114). Thus, further studies on the functional implication of GrB-producing Breg cells in the pathogenesis of pSS will provide new insight in understanding their target cells and immunopathology of pSS.

### IL-35-Producing Breg Cells

As a novel cytokine, IL-35 consists of p35 and EBI3, which is involved in mediating the regulatory functions of B cells. In mice with B cell-specific p35 (p35^-/-^) or EBI3 (Ebi3^-/-^) deficiency, exacerbated experimental autoimmune encephalomyelitis (EAE) was developed (44), indicating that IL-35-producing Breg cells restrain the pathogenesis of EAE. Moreover, IL-35-producing Breg cells have been shown to suppress Th1 and Th17 cells but induce Treg cell proliferation in the murine uveitis model (45). A recent study revealed higher levels of IL-35 expression in peripheral blood from SLE patients than healthy controls (115). Further investigations (116) have found that serum IL-35 concentrations are reduced in patients with lupus nephritis (LN) compared to those without LN and negatively correlated with disease activity, suggesting that IL-35 may take part in the development of lupus nephritis. Treatment of MRL/Lpr mice with IL-35 promotes the expansion of IL-10-producing Breg cells and ameliorates the disease progression (117). Although serum levels of IL-35 are reduced in pSS patients compared with healthy controls (13), increased EBI3^+^ B cells are observed, indicating that IL-35-producing Breg cells may play a role in the pathogenesis of pSS.

### Regulatory Plasma Cells

Recent studies have identified a novel subset of plasma cells expressing the inhibitory receptor LAG-3, which exert regulatory functions by secreting IL-10 in mice (46, 118). It has been shown that B cell receptor signaling is essential for the differentiation of LAG-3^+^CD138^hi^ regulatory plasma cells, since these plasma cells are not present in mice deficient for Btk. Moreover, Toll-like receptor signaling is critically involved in controlling IL-10 production in regulatory plasma cells (46). In addition, plasmablasts have also been reported to exert the regulatory function in an EAE mouse model, in which plasmablasts in the draining lymph nodes produce IL-10 to suppress autoimmune pathogenesis (102). Together, these studies provide further evidence on the expanding functional diversity of plasma cells in immunity and inflammation.

## Interactions Between Salivary Gland Epithelial Cells and B Cells

Lines of evidence suggest that the interactions between salivary gland epithelial cells (SGECs) and B cells may promote SS pathogenesis *via* multiple effector mechanisms (119). It has been shown that SGECs can drive B cell activation, differentiation and survival through the direct interaction and cytokine production (120, 121). In culture, SGECs from pSS patients promote B cell differentiation into mature B cell phenotypes (120). Moreover, the survival rates of B cells are also increased when cultured with SGECs from pSS patients (121). There is increasing evidence that SGECs may induce B cell differentiation in an indirect manner. It has been reported that SGECs can promote T follicular helper cell differentiation and IL-21 production (122), which may further enhance B cell hyperactivity in the salivary gland of pSS patients. Accordingly, the crosstalk between SGECs and B cells highlights a critical role of SG epithelial cells in SS pathogenesis.

## Conclusion

Available studies have indicated that various pathogenic B cell subsets contribute to the disease progression of pSS, while Breg cells alleviate the disease activities. However, there is evidence that B cell predominant phenotype does not involve all pSS patients, which warrants further studies on the pivotal roles of T cells and innate immune cell types in the pathogenesis of pSS (3, 123). Recent findings have provided new insight in understanding the pathogenic mechanisms of pSS and validated B cell-targeted therapy as future therapeutic options for patients with pSS (**Figure 1**). The major challenges in B cell-targeted therapy include the specific reduction of disease-related B cell subsets, instead of the complete depletion of the broader B cell population. In addition, Breg cell-based therapy may represent a promising clinical application for the therapeutic intervention. Further studies on the identification and functional characterization of novel B cell subsets in the pathogenesis of pSS will facilitate the development of new therapeutic strategies for Sjögren’s syndrome and other autoimmune diseases.

## Author Contributions

All authors contributed to the article and approved the submitted version.

## Funding

This work was supported by the National Natural Science Foundation of China (NSFC) (82071817, 82004171), Funding for Chongqing International Institute for Immunology (2020YJC10), Hong Kong Research Grants Council General Research Fund (17113319) and Theme-Based Research Scheme (T12-703/19R), the Fundamental Research Funds for Central Public Welfare Research Institutes (ZZ13-YQ-033-C1), Young Elite Scientist Sponsorship Program by CACM (CACM-2020-QNRC2-05) and HKU Seed Funding for Strategic Interdisciplinary Research Scheme.

## Conflict of Interest

The authors declare that the research was conducted in the absence of any commercial or financial relationships that could be construed as a potential conflict of interest.

The reviewer RL declared a shared affiliation with several of the authors, [XZ and HZ], to the handling editor at the time of review.
